# New York and London Mortality

**Published:** 1855-05

**Authors:** 


### NEW YORK AND LONDON MORTALITY.

People live longer in London than in any other large city in the world. And excepting epidemics, it is more healthy than the country, notwithstanding its dirty streets, its countless cesspools, its infecting cellars, and thousand other sources of disease.

| | | | | | |
|---|---|---|---|---|---|
| 31 | die each year out of | 1000 | under | 20 years | of age. |
| 10 | " | " | from | 20 to 40 | " |
| 23 | " | " | " | 40 to 60 | " |
| 72 | " | " | " | 60 to 80 | " |
| 224 | " | " | " | 80 upwards. | |

Estimating for the first week in July, 1854, one person a week dies in London out of seventeen hundred and ninety-six, while in a corresponding week in New York, one person dies a week, out of five hundred and eighty-five.

In London, each week, 1 dies out of every 1796.
In New York, " 1 " " 585.

According to this calculation, three times as many die in New York as in London, according to the population. Chemical analysis seems to point to the fact that smoke is a great absorbent of noxious emanations. London is enveloped in everlasting smoke. Can it be that the remarkable exemption of Pittsburgh from cholera is partially owing to this cause? It is worthy of observation.

---

Average Illness at Different Ages. — It is stated that between the ages of 20 and 30, each person has on an average nearly 7 days' illness a year; at 40 it is increased to 8 days; at 45 to 9; at 50 to 11 1-2; at 55 to 14; at 60 to 18 3-4; at 65 to 27 1-2; at 70 to 43 1-2; and 75 to 66; and at 80 to 97 1-2.

